# Multiple organ failure after trauma affects even long-term survival and functional status

**DOI:** 10.1186/cc6111

**Published:** 2007-09-04

**Authors:** Atle Ulvik, Reidar Kvåle, Tore Wentzel-Larsen, Hans Flaatten

**Affiliations:** 1 Department of Anaesthesia and Intensive Care, Haukeland University Hospital, Bergen, Norway; 2 Section for Anaesthesiology and Intensive Care, Department of Surgical Sciences, University of Bergen, Bergen, Norway; 3 Centre for Clinical Research, Haukeland University Hospital, Bergen, Norway

## Abstract

**Background:**

The aim of this study was to assess the incidence of organ failure in trauma patients treated in an intensive care unit (ICU), and to study the relationship between organ failure and long-term survival and functional status.

**Methods:**

This is a cohort study of all adult ICU trauma patients admitted to a university hospital during 1998 to 2003. Organ failure was quantified by the Sequential Organ Failure Assessment (SOFA) score. A telephone interview was conducted in 2005 (2 to 7 years after trauma) using the Karnofsky Index to measure functional status, and the Glasgow Outcome Score to measure recovery.

**Results:**

Of the 322 patients included, 47% had multiple organ failure (MOF), and 28% had single organ failure. In a Cox regression, MOF increased the overall risk of death 6.0 times. At follow-up, 242 patients (75%) were still alive. Patients with MOF had 3.9 times greater odds for requiring personal assistance in activities of daily living compared to patients without organ failure. Long-term survival and functional status were the same for patients suffering single organ failure and no organ failure. Complete recovery occurred in 52% of survivors, and 87% were able to look after themselves.

**Conclusion:**

Almost half of the ICU trauma patients had MOF. While single organ failure had no impact on long-term outcomes, the presence of MOF greatly increased mortality and the risk of impaired functional status. MOF expressed by SOFA score may be used to define trauma patients at particular risk for poor long-term outcomes.

## Introduction

Multiple organ failure (MOF) is the leading cause of morbidity and mortality in critically ill patients [1]. Recent studies report an incidence of MOF of between 5% and 25% for trauma patients admitted to the intensive care unit (ICU) [2-4].

MOF has been defined as progressive dysfunction of two or more organ systems following an acute threat to systemic homeostasis [5]. Several organ dysfunction scoring systems have been developed to describe and quantify organ dysfunction/failure in ICU patients [6-8]. The Sequential Organ Failure Assessment (SOFA) score quantifies and describes the evolution of organ dysfunction/failure over time [8], and has been validated in trauma patients [9]. Different derivations of the SOFA score have also been found to be related to short-term outcome, such as ICU mortality [1], but the relationship to long-term outcomes is more obscure.

The aim of the present study was to assess the incidence and severity of organ failure in trauma patients admitted to the ICU using the SOFA score. A further objective has been to study the relationship between organ failure and mortality and functional status 2 to 7 years after discharge from the ICU.

## Materials and methods

### Setting and study population

The study was performed in a mixed, 10-bed, closed ICU in a university hospital and included neurosurgical patients. Foreign citizens (*n *= 16) were not included due to difficulties in follow-up. The cohort study comprised 325 consecutive trauma patients above 18 years of age admitted to our ICU in the period 1998 to 2003. Three patients refused to participate in the study, leaving 322 patients for inclusion. A detailed analysis of survival for this cohort of trauma patients has been described elsewhere [10].

### The SOFA scoring system

The SOFA score assesses the function of six different organ systems: respiratory (partial arterial oxygen pressure (PaO_2_)/fraction of inspired oxygen (FiO_2_)), cardiovascular (blood pressure, vasoactive drugs), renal (creatinine and diuresis), hepatic (bilirubin), neurological (Glasgow Coma Score) and haematological (platelet count) [8]. During the ICU stay, each organ system was evaluated daily at 08.00 am using the most abnormal data from the preceding 24 h, and given a score from 0 (normal function) to 4 (most abnormal) according to the original definitions. Severe organ failure was defined as a SOFA score ≥3 in any organ system. MOF was defined as the occurrence of severe organ failure in two or more organ systems during the ICU stay, either on the same day or on different days.

### Data collection

The baseline characteristics age, sex, Simplified Acute Physiology Score (SAPS) II, and length of stay in the ICU were retrieved from our prospective ICU database [11]. In addition, data on respiratory, cardiovascular, and dialysis treatments were recorded from the database. Missing values were filled in from the patients' records as required. The SOFA score was completed in retrospect for the years 1998 and 1999, since SOFA scoring did not become a routine in our ICU until January 2000. Five patients had incomplete SOFA scores during their ICU stay, four for hepatic function and one for haematological function. These patients had a short ICU stay (range 0.2 to 1.6 days) and they did not suffer any failure in the other five organ systems. By default they were given a SOFA score of 0 for the organ system not assessed.

The Injury Severity Score (ISS) [12], an anatomical description of injury, has not been part of the routine ICU database, and was therefore calculated in retrospect using the 1990 version (update 1998) of the Abbreviated Injury Scale (AIS).

**Figure 1:**
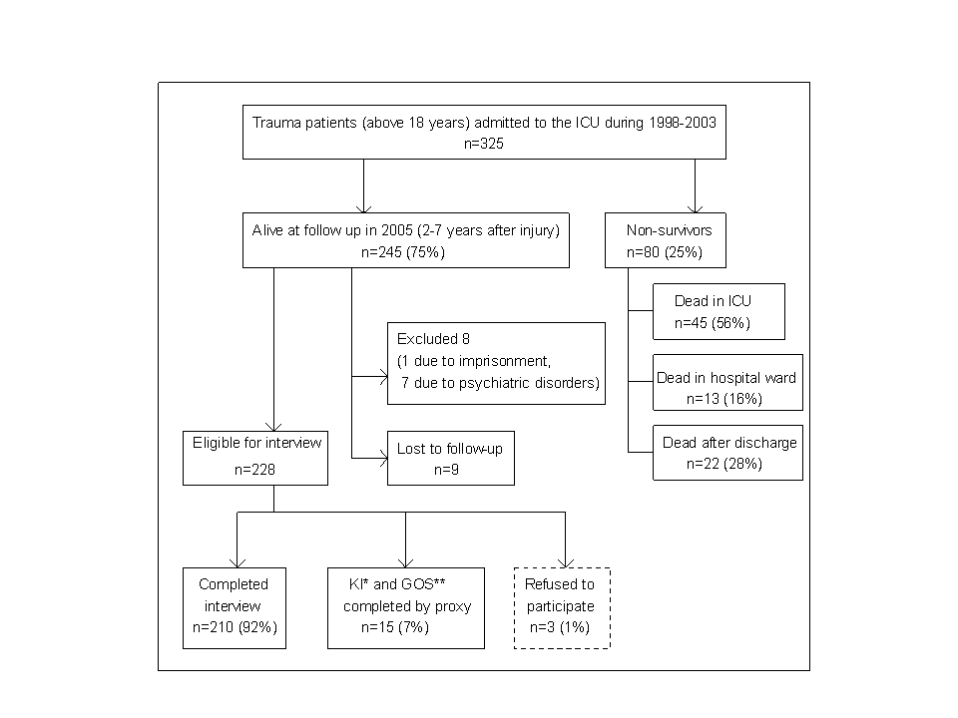
Enrolment and possible outcomes. GOS, Glasgow Outcome Score; ICU, intensive care unit; KI, Karnofsky Index.

Survival data were found in the Norwegian Population Registry. At follow-up in 2005, 245 patients were still alive (Figure 1). A letter was sent to the survivors with information about the study, underlining voluntary participation. Some weeks later the patients were interviewed on the telephone. Eight patients were not able to carry out a telephone interview, seven due to chronic psychiatric disorders and one due to imprisonment. These patients were excluded from further follow-up. Nine patients were lost to follow-up due to no permanent address. Three patients refused to participate in the study. Two physicians (AU and RK) performed the semi-structured interviews. The Glasgow Outcome Score [13] was used to measure recovery, and physical functional status was assessed by the Karnofsky Index [14]. In patients incapable of answering questions due to the trauma (*n *= 15), the Glasgow Outcome Score and Karnofsky Index were completed from information given by proxies.

### Statistical analysis

Based on the SOFA scoring system, the patients were categorized into no organ failure, severe single organ failure, or multiple organ failure, as described above. The baseline characteristics of these three groups were compared using exact chi-squared, Mann-Whitney and Kruskal-Wallis tests, and one-way ANOVA. The relationship between organ failure and long-term survival was analysed univariately by Kaplan-Meier survival statistics, using log rank tests for differences between groups, and multivariately by a Cox proportional hazards regression model. The proportional hazard assumption was checked based on Schoenfeld residuals [15]. Logistic regression was performed to analyze the association between organ failure and the Karnofsky Index score. All multivariate analyses were adjusted for age, sex, and severe head injury defined as a head AIS score ≥4.

Statistical analyses were performed using SPSS 12 (SPSS Inc, Chicago, IL, USA) and R (The R Foundation for Statistical Computing; Vienna, Austria). A *p *value < 0.05 determined statistical significance and all confidence intervals (CI) are 95%.

### Ethics

The study was approved by the regional ethical committee with acceptance of oral consent at the beginning of the telephone interview. No data are presented for the three patients who refused to participate in the study.

## Results

Of the 322 patients included, 81 had no organ failure, 91 had severe single organ failure, and 150 were in the MOF group. Comparison of baseline characteristics and selected ICU treatments for the three groups according to degree of organ failure are presented in Table 1. Patients with MOF were older and had a higher SAPS II and ISS, and a longer ICU stay compared to patients with no or only single organ failure. More patients in the MOF group had severe head injury.

**Table 1:** Baseline characteristics of critical care trauma patients categorized into no organ failure, single organ failure, and multiple organ failure

	No organ failure (*n *= 81; 25 percent)	Single organ failure^a ^(*n *= 91; 28 percent)	Multiple organ failure^b ^(*n *= 150; 47 percent)	*p *value
Male/female^c^	68/13	72/19	127/23	0.525
Mean age, years ± SD (range)^d^	37 ± 17 (18–82)	44 ± 19 (18–88)	47 ± 21 (18–88)	0.002
Median ISS (range)^e^	18 (8–41)	24 (4–57)	28 (4–54)	0.001
ISS <16 (percent)^f^	26 (32)	17 (19)	16 (11)	<0.001
Mean SAPS II ± SD (range)^d^	21 ± 9 (6–46)	32 ± 14 (12–69)	48 ± 15 (14–97)	<0.001
Mean length of stay in ICU, days ± SD (range)^e^	1.5 ± 1.1 (0.2–5.1)	4.1 ± 6.0 (0.1–48.3)	7.4 ± 6.7 (0.1–34.9)	<0.001
Severe head injury (percent)^f,g^	7 (9)	26 (29)	89 (59)	<0.001
Treatment in ICU^f^				
Respirator (percent)	22 (27)	71 (78)	144 (96)	<0.001
Vasopressor (percent)	1 (1)	12 (13)	132 (88)	<0.001
Dialysis (percent)	0	1 (1)	7 (5)	0.024

^a^SOFA score ≥3. ^b^SOFA score ≥3 in at least two organ systems. ^c^Exact chi-squared test. ^d^One-way ANOVA. ^e^Exact Kruskal-Wallis test. ^f^Exact Mann-Whitney test. ^g^Head Abbreviated Injury scale score ≥4. ICU, intensive care unit; ISS, Injury Severity Score; SAPS, Simplified Acute Physiology Score; SD, standard deviation.

The mechanisms of injury were mainly traffic accidents (52%) and falls (37%). The distribution of traffic accidents was: car (62%), motorcycle (16%), pedestrian (12%), bicycle (8%), other (2%). The trauma was a result of assault in 9 (3%), and of gunshot injury in 3 patients (1%).

In the single organ failure group, 57% had respiratory failure, 37% neurological failure, 3% cardiovascular failure, 2% renal failure, and 1% isolated liver failure. In the MOF group, 85% had cardiovascular failure, 79% respiratory failure, 73% neurological failure, 10% haematological failure, 9% renal failure, and 4% liver failure. In the MOF group, 56 patients (37%) had failures in three organ systems, and 15 (10%) had failures in more than three organ systems.

**Figure 2:**
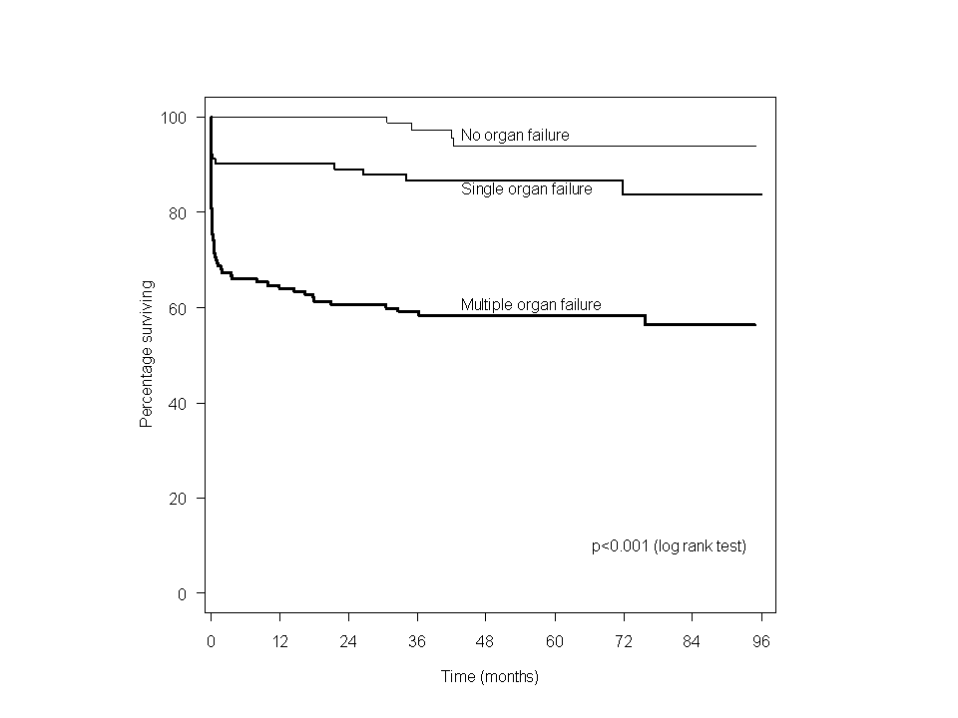
Survival of 322 trauma patients with no organ failure, single organ failure, and multiple organ failure treated in the intensive care unit.

At a median follow-up of 47 months (range 2 to 7 years) after discharge from the ICU, 242 (75%) of the 322 patients included were still alive. Overall mortality was significantly different in the three groups, and highest in the MOF group (Figure 2). Taking the substantial initial mortality into consideration, we performed a Kaplan-Meier survival analysis excluding those who did not survive until 30 days; MOF patients still had a higher long-term mortality (*p *= 0.006, log rank test).

Cox regression analyses with adjustment for age, sex, and severe head injury showed that the presence of MOF increased the risk of death 6.03 times (95% CI 2.46 to 17.14) compared to patients with no organ failure. Single organ failure increased the risk of death 2.46 times (95% CI 0.79 to 7.62); although clinically relevant, this was not statistically significant (*p *= 0.119). There were significant deviations from the proportional hazard assumptions for the organ failure contrasts (no organ failure, single organ failure, MOF; *p *≤ 0.016) and sex (*p *= 0.017). Schoenfeld residual plots showed, however, that the deviations were due to a few data points in the last (organ failure contrasts) and first (sex) part of the follow-up.

As a *post hoc *sensitivity analysis we repeated the Cox regression replacing the categorized organ failure variable by, respectively, admission SOFA score, maximum SOFA score, delta SOFA score (the difference between maximum score and SOFA score at ICU admission), and ISS, with the same adjustment variables. In these regressions, admission and maximum SOFA score (*p *< 0.001) were significantly related to long-term survival, while ISS and the delta SOFA score were not. For both admission and maximum SOFA score, the hazard ratio for about a nine point difference was equal to the hazard ratio for MOF versus no organ failure.

While 27% of patients in the MOF group died in the ICU, all patients without severe organ failure survived until hospital discharge (Table 2).

**Table 2:** Mortality among trauma patients treated in the intensive care unit

	**No organ failure (*n *= 81)**	**Single organ failure (*n *= 91)**	**Multiple organ failure (*n *= 150)**
Overall mortality (%)	4 (5)	13 (14)	63 (42)
Place of death			
ICU	0	5	40
Hospital ward	0	4	9
After hospital discharge	4	4	14

Significant differences between the multiple organ failure group and the two other groups, *p *< 0.001; no significant difference between the single and no organ failure groups, *p *= 0.059; Cox proportional hazards regression. ICU, intensive care unit.

Table 3 shows the Glasgow Outcome Score and Karnofsky Index in 225 of the 228 eligible survivors. Overall, 90% achieved either good recovery or moderate long-lasting disability according to the Glasgow Outcome Score. In the MOF group, 17% were classified as severely disabled and 4% as persistent vegetative.

**Table 3:** Recovery and functional status 2 to 7 years after discharge from the intensive care unit

	No organ failure (*n *= 71)	Single organ failure (*n *= 73)	Multiple organ failure (*n *= 81)
**Glasgow Outcome Score (percent)**^a^			
Good recovery	44 (62)	37 (51)	36 (44)
Moderate disability	26 (37)	31 (42)	28 (35)
Severe disability	1 (1)	4 (6)	14 (17)
Persistent vegetative	0	1 (1)	3 (4)
			
**Karnofsky Index**^b^			
30. Severely ill, hospitalized	0	2	2
40. Disabled, requires special assistance	1	1	4
50. Unable to work, requires much assistance	0	2	8
60. Unable to work, in need of occasional help	2	0	7
70. Unable to work, but able to look after self	11	14	11
80. Continues most activities with some effort	15	24	17
90. Minor symptoms and limits on activities	28	20	24
100. No symptoms, no limits on activities	14	10	8

^a^*p *< 0.001; exact (using Monte Carlo) linear by linear association test. ^b^*p *< 0.001; logistic regression for Karnofsky Index above 60, adjusted for age and sex.

Of these 225 survivors, 87% had a Karnofsky Index above 60, which corresponds to being able to live independently without assistance from others. Of the 144 patients without MOF, 94% had a Karnofsky Index above 60, and in the MOF group, 74% had a Karnofsky Index above 60. Using logistic regression with adjustment for age, sex, and severe head injury, organ failure was significantly related to this dichotomised Karnofsky index (*p *= 0.042). Patients with MOF had an odds ratio of 3.88 (95% CI 0.99 to 15.21) for requiring assistance from others in activities of daily living more than 2 years after the trauma compared with patients with no organ failure. There was no significant difference in Karnofsky Index score between the no organ failure group and the single organ failure group (*p *= 0.794).

Of the 210 patients who completed the interview, 155 were full-time workers prior to trauma, three were part-time workers, 16 were students, 8 were unemployed, 11 lived on Social Security, and 17 were pensioners. At interview, 83 were full-time workers, 20 part-time workers, 18 students, 5 unemployed, 65 living on Social Security, and 19 were retired. Of the 171 full-time workers or students prior to trauma, 97 (57%) were still a full-time employee or student 2 to 7 years after discharge from the ICU. Of the 74 patients no longer employed full-time, 68 reported that they had changed work status due to the trauma.

## Discussion

In the present study, multiple organ failure occurred in 47% of the patients, and was significantly associated with long-term survival and functional status. Of the 322 patients, 75% were still alive at follow-up 2 to 7 years after discharge from the ICU. Of the survivors, good recovery and moderate disability were found in 52% and 38%, respectively, according to the Glasgow Outcome Score. Using the Karnofsky Index, 87% were able to live independently without assistance from others in activities of daily living.

MOF is a major cause of morbidity after severe injury [4]. Recent studies of ICU trauma populations have found an incidence of MOF of between 5% and 25% [2-4]. In the present study, almost half of the trauma patients developed MOF. It is likely that the case-mix and differences in ICU admission policy can explain most of this large variation in reported incidence of MOF. In addition, the application of different scoring systems for assessment of MOF makes direct comparison difficult. In our hospital, trauma patients without severe organ failure are usually treated outside the ICU, and only 25% of the ICU trauma patients had no severe organ failure.

Consistent with previous literature [1,8], we defined organ failure according to the SOFA score definitions. Several multiple organ dysfunction scoring systems have been developed, but the SOFA score and the Multiple Organ Dysfunction Score [7] are the most commonly applied. The SOFA score has been validated in trauma patients [9]. In a recent study of patients with brain injury, the SOFA scoring system had superior discriminative ability and stronger association with hospital mortality and unfavourable neurological outcome compared with the Multiple Organ Dysfunction Score [16].

A major finding in our study was the relationship between MOF and long-term outcomes after severe trauma. From ICU admittance and up to 7 years post injury, patients suffering MOF had an overall mortality of 42%. Severe head injury has been reported to be the leading cause of both early and late deaths after trauma [4,10,17]. Therefore, in the present study, we included severe head injury as an adjustment variable in the regression analyses. Although MOF no longer is considered a primary cause of death, we found that the presence of MOF increased the risk of death by six times compared to patients without organ failure. Single organ failure did not significantly increase the risk of death.

We also found a strong relationship between the degrees of organ failure immediately after injury, and late functional status. In a multivariate analysis, adjusted for age, sex, and severe head injury, patients with MOF had four times greater odds of requiring assistance from others in activities of daily living more than 2 years after trauma compared to trauma patients without organ failure. There was no significant difference regarding self-care among patients with no organ failure and those with a single organ failure.

An association between SOFA score and different hospital outcomes has been reported [1,9,16,18]. The more sophisticated derived measurements of the SOFA score, that is, the maximum SOFA score and the delta SOFA score (the difference between maximum score and SOFA score at ICU admission), were used in these studies. They showed that ICU mortality, hospital mortality, and length of stay in the ICU all increased with increasing degree of organ failure. However, the relationship between organ failure, quantified by SOFA score, and long-term outcome, has not been documented previously. It is interesting, therefore, that the simple usage of the SOFA score to categorise trauma patients into MOF or not enables us to identify patients at risk of both impaired long-term survival and impaired long-term functional status.

Functional status is one of the most important outcome measures of critical care because it describes the level of independence enjoyed by the patient [19]. Functional status can be objectively assessed by a third party, in contrast to the subjective quality of life assessments, which also include an element of patient satisfaction. The Karnofsky Index is a system for general classification of the patient's performance status [14], and has been applied to ICU survivors to measure functional outcome [20]. The scaling takes account of the presence of symptoms, the ability to work, physical activity, and self-care. In our study, 87% of the survivors were able to look after themselves with no need for assistance in their daily lives. A straightforward comparison of functional status with other ICU trauma populations is difficult because of the difference in outcome measurement instruments used. In addition, functional outcome is frequently and incorrectly used interchangeably with quality of life [19]. In a study of a general ICU population, 25% of the patients required assistance from others in daily life at follow-up 8 months after ICU discharge [20].

The Glasgow Outcome Score has been recommended as a rough overall assessment for all trauma patients [21]. In our study with assessment of outcome up to 7 years after severe trauma, only 52% of the survivors achieved good recovery with resumption of normal life despite minor deficits. Thus, half of the patients still suffered some kind of disability. Although we included all trauma patients admitted to the ICU independent of ISS, the proportion of patients experiencing good recovery after 2 years was lower in our study compared to the 70% to 77% reported by others [22,23]. The reason for this disparity might be differences in patient selection. In these studies only patients with an ISS ≥16 were included regardless of ICU admission.

The present study is a single centre study. Differences in ICU admission policies and case-mix may complicate direct comparison with other studies. The trauma patients in this study were predominately victims of traffic accidents and falls. A further limitation is that our findings may not be fully applicable to ICU trauma populations with a greater proportion of other mechanisms of injury, for example, gunshots and penetrating injuries.

## Conclusion

Almost half of the ICU trauma patients had MOF. While single organ failure had no impact on long-term outcomes, the presence of MOF greatly increased the mortality and the risk of impaired functional status. More than 2 years after severe trauma only half of the ICU survivors had fully recovered with resumption of normal life. However, most of the patients were able to look after themselves. This study documents that MOF expressed by SOFA score may be used to define trauma patients at particular risk of poor long-term outcomes.

## Key messages

• Half of adult trauma patients in our ICU suffered MOF.

• MOF was strongly associated with increased long-term mortality and impaired functional status.

• Although most trauma ICU survivors were able to look after themselves, only half of the patients had fully recovered more than 2 years post-injury.

• MOF expressed by SOFA score can define trauma patients at particular risk of poor long-term outcomes.

## Abbreviations

AIS = Abbreviated Injury Scale; CI = confidence interval; ICU = intensive care unit; ISS = Injury Severity Score; MOF = multiple organ failure; SAPS = Simplified Acute Physiology Score; SOFA = Sequential Organ Failure Assessment.

## Competing interests

The authors declare that they have no competing interests.

## Authors' contributions

AU was involved in the design of the study, in the acquisition, analysis and interpretation of data, and drafted the manuscript. RK helped to design the study, and participated in the acquisition of data. TW-L participated in the design of the study, and analysis and interpretation of data. HF helped to design the study, and participated in the acquisition of data. All authors revised the manuscript critically. All authors have read and approved the final manuscript.
